# The Current Role of Endourologic Management of Renal Transplantation Complications

**DOI:** 10.1155/2013/246520

**Published:** 2013-08-19

**Authors:** Brian D. Duty, Michael J. Conlin, Eugene F. Fuchs, John M. Barry

**Affiliations:** ^1^Department of Urology, Oregon Health & Science University, 3033 SW Bond Ave, CH10U, Portland, OR 97239, USA; ^2^Department of Urology, Oregon Health & Science University/Portland VA Medical Center, 3033 SW Bond Ave, CH10U, Portland, OR 97239, USA; ^3^Departments of Urology and Surgery, Oregon Health & Science University, 3033 SW Bond Ave, CH10U, Portland, OR 97239, USA

## Abstract

*Introduction*. Complications following renal transplantation include ureteral obstruction, urinary leak and fistula, urinary retention, urolithiasis, and vesicoureteral reflux. These complications have traditionally been managed with open surgical correction, but minimally invasive techniques are being utilized frequently. *Materials and Methods*. A literature review was performed on the use of endourologic techniques for the management of urologic transplant complications. *Results*. Ureterovesical anastomotic stricture is the most common long-term urologic complication following renal transplantation. Direct vision endoureterotomy is successful in up to 79% of cases. Urinary leak is the most frequent renal transplant complication early in the postoperative period. Up to 62% of patients have been successfully treated with maximal decompression (nephrostomy tube, ureteral stent, and Foley catheter). Excellent outcomes have been reported following transurethral resection of the prostate shortly after transplantation for patients with urinary retention. Vesicoureteral reflux after renal transplant is common. Deflux injection has been shown to resolve reflux in up to 90% of patients with low-grade disease in the absence of high pressure voiding. Donor-gifted and de novo transplant calculi may be managed with shock wave, ureteroscopic, or percutaneous lithotripsy. *Conclusions*. Recent advances in equipment and technique have allowed many transplant patients with complications to be effectively managed endoscopically.

## 1. Introduction

The incidence of end-stage renal disease in the United States is 360 individuals per million population per year, and over 870,000 people currently live with the disease [1]. More than 40 billion dollars were allocated to end-stage renal disease (ESRD) in 2009 [1]. By the end of 2009 nearly 400,000 ESRD patients were managed with dialysis and 172,553 had functioning kidney transplants [1]. The 5-year survival rate for patients with a functioning renal transplant is markedly better than that for individuals on dialysis (85.5% versus 35.8%) and the costs of dialysis are nearly three times those of transplantation [1].

Despite the survival and cost benefit, a significant number of kidney transplant recipients experience urologic complications. Streeter et al. reviewed 1535 consecutive renal transplants and noted the following complications: ureteral obstruction (3.0%), urinary leak (2.9%), bladder outlet obstruction (1.1%), and obstructive ureteral calculi (0.3%) [2]. Mean recipient age was 43 years. Vesicoureteral reflux and urinary fistula have also been reported [3, 4]. A study of 206 pediatric recipients found obstruction and/or urinary extravasation in 7.6%, reflux in 10.6%, and graft loss in 17% of patients [5]. These complications have traditionally been managed with open surgical procedures. Advances in equipment have allowed an increasing number of patients to be treated with less invasive techniques. This paper will review the contemporary role of endourological management of renal transplant complications. 

## 2. Materials and Methods

A PubMed database search was performed with the terms “transplant,” “ureteral stricture,” “ureteral obstruction,” “urine leak,” “urinary fistula,” “bladder outlet obstruction,” “urinary retention,” “vesicoureteral reflux,” “stone,” “calculi,” “shock wave lithotripsy,” “ureteroscopy,” and “percutaneous nephrolithotomy.” Only full-text articles published in English language were reviewed. References of selected articles were also evaluated for pertinent manuscripts. Case reports were excluded. A comprehensive review of the literature was performed, but a systematic review of the data/meta-analysis was not possible. 

## 3. Results and Discussion

Few studies pertaining to the endourologic management of urologic transplant complications have been published. The majority of manuscripts have been small, heterogeneous case series, with highly variable follow-up, if reported at all. In addition, the definition and assessment of treatment success vary greatly between studies. 

Each section begins with a review of the incidence and etiology of the detailed complication. The limited data on endourologic management is then reported. When possible, case series have been combined as summarized in Table 1. Management recommendations based upon review of the literature are provided. However, these recommendations are entirely grounded in Level IV data. Until larger, well-designed, prospective studies are performed; recommendations will unfortunately be based in large part upon “expert opinion.” 

### 3.1. Ureteral Obstruction

Ureteral stricture formation is one of the most common complications following transplantation. The ureterovesical anastomosis is most commonly involved. The reported incidence varies from 1% to 4.5% [6]. Stricture formation usually results from ureteral devascularization, but extrinsic obstruction (e.g., lymphocele, fibrosis) and errors in surgical technique may also occur. 

Risk factors include donor age greater than 65, more than two allograft renal arteries, prolonged cold ischemia time, and a stentless anastomotic technique [7]. Gonadal vessel preservation, retrieval modality (open versus laparoscopic), and implantation technique (intra-versus extravesical) have not been associated with stricture formation [7]. 

Asymptomatic deterioration of renal function is the most common presentation. Renal insufficiency usually develops early in nonstented individuals and following stent removal in patients with stented anastomoses. Workup begins with either ultrasonography (US) or computed tomography (CT). CT is advantageous because it can characterize the presence and extent of hydroureter and can identify sources of extrinsic and intrinsic obstruction. 

Endourologic techniques have been traditionally used to decompress the collecting system prior to undertaking open ureteral reconstruction. Retrograde ureteral stent placement is often challenging. As a result, many consider percutaneous nephrostomy tube placement as the first-line intervention for ureteral obstruction. 

More recently, endourologic management has been undertaken as a primary treatment modality. Options include balloon dilation or endoureterotomy. The latter may be accomplished with a cold knife, Acucise (Applied Medical Systems, Laguna Hill, CA, USA) balloon-cutting catheter or Holmium laser. Each may be performed in an antegrade or retrograde fashion; however, proceeding from the area of dilation (i.e., antegrade approach) is often easier. 

Several contemporary series of primary balloon dilation have been published including a total of 94 patients [8–11]. At a mean follow-up of 37.3 months (range 17 to 78 months), the reported success rate was 51% (range 44% to 62%). Repeat dilatation was effective only 25% of the time [5]. One series found dilation within 3 months of transplantation to be associated with improved success (74% versus 44%) [8].

The Acucise balloon-cutting device was first introduced in 1993 and has been used primarily to treat ureteropelvic junction obstructions. However, a few small studies have detailed its use for ureterovesical strictures following transplantation [12, 13]. A total of 21 patients were treated with follow-up ranging from 13 to 27 months (mean 19 months). Success rates ranged from 60% to 100%, with an average of 78%. Despite reasonable success rates, the technique has fallen out of favor because of bleeding associated with blind ureteral incision. 

Direct vision endoureterotomy has become the preferred endourologic treatment option because it is more effective than balloon dilation and safer than blind ureteral incision. The endoureterotomy may be made with a cold knife, electrocautery, or Holmium laser. The overall success rate from five series was 79% (range 63% to 100%) at a mean follow-up of 29 months [6, 14–17]. However, these were small, heterogeneous studies that included all three techniques performed from both antegrade and retrograde approaches. 

Ureterovesical anastomotic stricture is the most common long-term urologic complication following renal transplantation. Although traditionally managed in an open fashion, endourologic treatment may be undertaken in select patients (early presentation, partial obstruction, and distal strictures less than 1 cm) with direct vision endoureterotomy being the preferred technique. 

### 3.2. Urinary Leak/Urinary Fistula

With an incidence ranging from 1.2% to 8.9%, urine leak is the most common early urologic transplant complication [4]. As with stricture formation, devascularization of the distal ureter is a major risk factor. Technical error may also result in a nonwatertight anastomosis. 

The ability of routine stent placement during transplantation to prevent urine leak is controversial. A randomized study of 194 patients found the urinary leak rate to be higher in unstented (6%) compared to stented patients (1%) [18]. In contrast, a randomized study of 280 patients did not detect a difference in ureteral obstruction or leak rates between stented (3.5%) and unstented (6.6%) recipients (*P* = 0.23) [19].

Initial management includes Foley catheter and percutaneous nephrostomy tube placement. An antegrade ureteropyelogram will determine the location and severity of the leak, which is most commonly at the ureterovesical anastomosis. Significant urinomas can be percutaneously drained to reduce the risk of infection and extrinsic ureteral obstruction. Definitive management has traditionally consisted of open reimplantation with resection of any devitalized ureter. Patients with urethrocutaneous or vesicocutaneous fistula can additionally undergo resection of the fistula tract with omental interposition. 

Several small series have evaluated primary endourologic management [20–24]. Among 65 reported patients, 62% (range 36% to 87%) were successfully managed at a mean follow-up of 35 months (range 24 to 67 months). Although less invasive, endourologic treatment is not always less morbid. In the series with the highest success rate, two patients died of sepsis and three grafts were lost [20]. 

Contraindications to endourologic management include proximal or extensive contrast extravasation and inability to place an antegrade ureteral stent. Primary endourologic treatment requires maximal decompression with a nephrostomy tube, ureteral stent, and Foley catheter. Periodic ureteropyelograms are performed. Once the leak has resolved, the nephrostomy tube and Foley catheter are removed and the ureteral stent is continued for another 4 to 6 weeks. Close follow-up is required following stent removal because stricture formation may result in silent obstruction and renal failure. 

### 3.3. Bladder Outlet Obstruction

Bladder outlet obstruction is of particular concern in the transplant population. Elevated voiding pressures promote urinary leak early in the postoperative period and chronic vesicoureteral reflux. Age greater than 60 and need for dialysis lasting more than 120 days have been associated with urinary retention following transplantation [25]. Consequently, the incidence of retention is likely to increase because ESRD rates are rising most quickly among individuals older than 65 years [26].

Streeter and colleagues' review of 1535 patients undergoing renal transplantation found a retention rate of 1.2% [2]. Five cases were attributed to benign prostatic hyperplasia (BPH), 5 to bladder neck contracture, 4 to urethral stricture, and 3 were idiopathic. Tsaur et al. compared the incidence of voiding dysfunction in male recipients 60 years or older to those between the ages of 40 and 59 [27]. Voiding dysfunction occurred in 27% of older recipients compared to 19% in the younger group. Prostatic hyperplasia was the most frequent cause in both cohorts, but it was more common in the older group (25% versus 12%; *P* = 0.009). Within the older cohort, 81% of patients were managed by transurethral prostate resection and the remainder was treated with alpha-blockers. No perioperative complications were noted and none of the patients had retention following prostate resection. 

Another study reported no complications and a 100% success rate in 11 patients treated with transurethral resection of the prostate (TURP) in the early postoperative period (one to two weeks following transplantation) [28]. In contrast, Reinberg's review of patients undergoing TURP within 10 days of transplantation found a major complication rate of 25%, and one mortality [29]. However, this was a much older study with only 8 patients. 

The optimal timing of resection has not been determined but waiting at least a month after transplantation to ensure the ureterovesical anastomosis has had time to fully heal loses little. Outlet procedures should not be performed prior to transplantation because of the increased risk of urethral stricture formation in oliguric patients [27]. Lastly, urodynamics should be considered in patients with concomitant neurologic conditions or severe diabetic neuropathy. 

### 3.4. Vesicoureteral Reflux

Depending upon the ureteroneocystostomy technique used, the incidence of vesicoureteral reflux following transplantation can be as high as 50% to 86% [30, 31]. The high rate of vesicoureteral reflux (VUR) is believed to be largely due to surgical technique, with many surgeons performing a wide open anastomosis in an effort to prevent stricture formation. 

The significance of reflux in adults after transplantation has been debated. Favi et al. compared 15 recipients with VUR to 22 without [32]. Each patient had at least one urinary tract infection (UTI) per year and was more than 2 years out from transplant. The authors found no difference in the number of infections per year, serum creatinine, graft, and overall survival. However, none of the patients had grade IV or V reflux and 26% had only Grade I. A similar study including patients with Grade IV reflux also found no difference in UTI rate or graft dysfunction, but follow-up did not extend beyond 12 months [33]. In a study with follow-up out to five years, recipients with reflux were more likely to develop urosepsis and hypertension [31].

Regardless of the aforementioned controversy, most clinicians agree that patients with recurrent UTIs or UTI-associated sepsis in the setting of high-grade VUR require surgical therapy. Treatment has traditionally been accomplished via open reimplantation or ureteroureterostomy to the native ureter. These are invasive procedures with the potential risks of anastomotic stricture, urinary fistula, and ureteral necrosis. 

In 1995, Stenberg and Lackgren reported the use of a novel dextranomer/hyaluronic acid copolymer, Deflux, for the endoscopic treatment of VUR [34]. Unlike other bulking agents, Deflux rarely incites an immunogenic foreign body response and has minimal particle migration. It has been used extensively in children with primary VUR with success rates up to 85% [35]. The first Deflux series for transplant-associated VUR was published in 2007 [36]. Four women with deteriorating renal function attributed to VUR were treated. One patient was cured following the first injection and two after a second injection. The remaining patient developed a ureteral stricture and required open repair. 

The two largest Deflux series to date include a total of 45 patients [3, 37]. The Pichler et al. study included 19 patients with at least three UTIs per year and documented VUR. The average number of infections per year was reduced from 4.89 to 1.31. Reflux resolution was noted in 57.9% of patients after the first injection and 78.9% after a second injection. Two patients developed ureteral obstruction requiring temporary percutaneous drainage. Yucel et al. reported an overall success rate of 53.8% in 26 patients [37]. Recipients with Grades I and II reflux had a success rate of 90%, and only 31% of individuals with Grades III and IV reflux were cured. The authors also evaluated subureteral versus intraureteral injection techniques and found no difference in treatment success in either low- or high-grade VUR patients. 

In summary, the incidence of VUR following renal transplantation is high, but its long-term impact on allograft function remains controversial. Nevertheless, patients with recurrent UTIs and VUR should be considered for correction. Success rates following Deflux injection in transplant patients are inferior to those with primary VUR. This is most likely due to the ectopic location and short intramural tunnel of the allograft ureter. Endoscopic management may be contemplated in patients with low-grade reflux. Recipients with Grades III and IV disease and/or high voiding pressures are better managed with open surgical correction. 

### 3.5. Transplant Calculi

The reported incidence of allograft stone disease varies significantly among different series. However, a review of 42,096 transplant recipients within the United States Renal Data System found an incidence of 0.11% and 0.15% for men and women, respectively [37]. Allograft stones may be acquired from the donor or developed following transplantation. The incidence of donor-gifted stones in a contemporary study was 0.64% [38]. The average time to presentation after surgery varied from 1.6 to 3.6 years [39].

Transplant recipients have a variety of anatomic and physiologic factors that predispose them to stone formation. Secondary VUR, partial ureteral obstruction, and retained suture material promote stone development. Renal tubular acidosis and tertiary hyperparathyroidism are common among kidney transplant recipients, which result in hypercalciuria and hypocitraturia. Calcineurin inhibitor (cyclosporine and tacrolimus) use is associated with hyperuricosuria.

Obstructive ureteral calculi do not result in renal colic because of allograft denervation. Vague lower abdomen discomfort may result from peritoneal irritation from a hydronephrotic collecting system. Patients frequently present in a delayed fashion with decreased urine output and renal failure. Nonobstructive intrarenal stones may result in recurrent UTIs. The allograft is often more prominent on physical exam due to collecting system dilation. A renal US should be performed in recipients with acute renal insufficiency. If hydronephrosis is present, a noncontrast CT will better characterize ureteral and surrounding anatomy. 

Conservative management of ureteral calculi may be undertaken if the stone is small and there is little to no deterioration in renal function. However, the threshold to intervene should be much lower than in non-transplant patients because of the recipient's immunosuppression and solitary functioning kidney. 

Patients presenting with significant renal insufficiency are best initially managed with urinary decompression by either a percutaneous nephrostomy or ureteral stent. Stent placement is often challenging due to the anterolateral location of the reimplanted ureter, and nephrostomy tube placement is often the most expeditious means of decompression. 

Definitive treatment is performed once the patient's renal function has recovered. Treatment options include shock wave, ureteroscopic, and percutaneous lithotripsy. No level one data exists in the transplant population to guide decision making. Nevertheless, most authors consider extracorporeal shockwave lithotripsy (ESWL) or ureteroscopic lithotripsy (URS) a reasonable option for stones less than 1.5 cm and percutaneous nephrolithotomy (PCNL) for larger calculi. 

ESWL has the advantage of being the least invasive treatment modality, but stone targeting may be difficult due to the allograft's pelvic location. Several small series have confirmed ESWL as a viable treatment option with success rates ranging from 87% to 100% [40–43]. However, the need for repeat ESWL is common (8 of 13 patients in the Challacombe et al. series) [42]. Close follow-up is mandatory in unstented patients because of the risk of ureteral obstruction during fragment passage. 

Compared to ESWL, URS has the advantage of being able to remove stone fragments. Retrograde access can be challenging and often requires ancillary instruments such as the Kumpe and Cobra Access Catheters (Cook Medical, Bloomington, IN, USA). If a nephrostomy tube had been previously placed, preoperative ureteral stent internalization or antegrade wire placement into the bladder at the time of surgery will facilitate retrograde access. 

Hyams et al. reported URS in 12 patients [44]. Five had previously undergone nephrostomy tube placement. Eleven patients were rendered stone-free. Two recipients experienced complications (nephrocutaneous fistula and ureteral stent encrustation). Basiri et al. reported a lower success rate (78%) in 15 patients managed in a retrograde fashion [45]. Urinary leak and UTI occurred in two patients. 

Percutaneous nephrolithotomy is the treatment of choice for stones larger than 1.5 cm. The procedure is performed in the supine position and access is obtained through an anterior calyx. Although the collecting system is often just under the abdominal wall musculature, a preoperative CT scan or intraoperative US should be performed to rule out overlying bowel. Tract dilation can be a challenge because of significant scar tissue around the allograft. The three largest contemporary series included a total 35 patients [46–48]. The overall stone-free rate was 88.5% with only three reported complications (postoperative sepsis, gastrointestinal bleed, and herpes esophagitis). 

## 4. Conclusions

The vast majority of patients undergoing renal transplantation enjoy a dramatic improvement in quality of life and overall survival with minimal morbidity. Until recently, the few patients who experienced a urologic complication were treated with open surgical correction. Fortunately, advances in equipment and technique have allowed many of these patients to be effectively managed in an endoscopic manner with minimally invasive techniques.

## Figures and Tables

**Table 1:** Summary of case series by transplant complication and endourologic treatment modality.

Complication	Treatment modality	No. of series	No. of patients	Mean success rate (range)
Ureteral obstruction	Balloon dilation	4	94	51% (44 to 62%)
	Acucise endoureterotomy	2	21	78% (60 to 100%)
	Direct vision endoureterotomy	5	17	79% (63 to 100%)
Urinary leak/fistula	Maximal urinary decompression	6	65	62% (36 to 87%)
Bladder outlet obstruction	Transurethral resection of prostate	3	54	98% (88 to 100%)
Vesicoureteral reflux	Deflux injection	3	49	53% (25 to 58%)
Transplant calculi	Shock wave lithotripsy	4	57	88% (62 to 100%)
	Ureteroscopic lithotripsy	2	27	85% (78 to 92%)
	Percutaneous nephrolithotomy	3	35	89% (77 to 100%)

## Abbreviations

BPH: Benign prostatic hyperplasia
CT: Computed tomography
ESRD: End-stage renal disease
ESWL: Extracorporeal shockwave lithotripsy
PCNL: Percutaneous nephrolithotomy
TURP: Transurethral resection of the prostate
URS: Ureteroscopic lithotripsy
US: Ultrasonography
UTI: Urinary tract infection
VUR: Vesicoureteral reflux.
